# Dynamic activation and dual roles of SOX9^+^ hepatocytes in liver regeneration under acute and chronic injury by single-cell transcriptomics

**DOI:** 10.3389/fcell.2026.1743286

**Published:** 2026-02-18

**Authors:** Junwei Zhang, Yiyao Xu, Xin Lu

**Affiliations:** 1 Department of Liver Surgery, State Key Laboratory of Complex Severe and Rare Diseases, Peking Union Medical College Hospital, Chinese Academy of Medical Sciences and Peking Union Medical College, Beijing, China; 2 Chinese Academy of Medical Sciences Oxford Institute, University of Oxford, Oxford, United Kingdom

**Keywords:** liver injury, liver regeneration, partial hepatectomy, single-cell RNA sequencing, SOX9-positive hepatocytes

## Abstract

**Background:**

SOX9^+^ hepatocytes, marked by progenitor-like features, have been implicated in both liver regeneration and fibrosis. This study systematically characterized the temporal and contextual roles of SOX9^+^ hepatocytes during acute and chronic liver injury using integrated single-cell transcriptomics.

**Methods:**

Publicly available single-cell RNA sequencing (scRNA-seq) datasets derived from mouse liver models of partial hepatectomy (PHx) and acetaminophen-induced acute liver injury (APAP), as well as human liver tissues with nonalcoholic fatty liver disease (NAFLD), were analyzed. Data were processed using Seurat and Harmony for batch correction. Pathway enrichment analysis was performed using the Kyoto Encyclopedia of Genes and Genomes (KEGG) and gene set enrichment analysis (GSEA).

**Results:**

Under PHx, SOX9^+^ hepatocytes exhibit transient metabolic activation associated with early regenerative responses and hepatocyte proliferation. In the APAP model, SOX9^+^ hepatocytes display a biphasic activation pattern, characterized by an early response to stress- and cytokine-associated signals followed by restoration of metabolic balance. During NAFLD progression, SOX9^+^ hepatocytes progressively expand and acquire transcriptional programs associated with inflammatory signaling and wound-healing processes.

**Conclusion:**

Our findings highlight SOX9^+^ hepatocytes as a dynamic and context-dependent hepatocyte subpopulation associated with adaptive metabolic responses during regeneration and altered inflammatory and wound-healing–related programs under chronic injury.

## Highlights

• Single-cell analysis reveals dynamic SOX9^+^ hepatocyte states across liver injury.
• SOX9^+^ hepatocytes show transient activation after partial hepatectomy.
• SOX9^+^ hepatocytes expand and adopt inflammatory features during NAFLD/NASH.
• SOX9^+^ hepatocytes represent a context-dependent hepatocyte subpopulation.

## 1 Introduction

The liver is the only solid organ in mammals capable of fully restoring its mass and function after injury (Bangru and Kalsotra, 2020). Following partial hepatectomy (PHx) or acute toxic insult, residual mature hepatocytes proliferate to compensate for tissue loss and re-establish normal liver architecture (Michalopoulos, 2021). However, when hepatocyte proliferation is insufficient or impaired, hepatic progenitor cells (HPCs) are activated to assist in tissue repair. Some studies suggest that cholangiocytes can transdifferentiate into biphenotypic progenitor-like cells (Manco et al., 2019; Pu et al., 2023), whereas others have demonstrated that, under chronic injury, hepatocytes themselves acquire plasticity and adopt hybrid phenotypes (Gribben et al., 2024). Despite decades of research, the definition and origin of HPCs remain controversial and require further investigation (Li et al., 2023).

SOX9, a transcription factor essential for biliary and progenitor cell differentiation, plays a central role in both liver development and adult tissue homeostasis (Kawaguchi, 2013). Increasing evidence points to the presence of SOX9^+^ hepatocytes, a progenitor-like subpopulation capable of contributing to tissue repair (Shao et al., 2022; Han et al., 2025). These cells exhibit bipotent differentiation potential, coexpressing hepatocyte and cholangiocyte markers during regeneration or chronic injury, and can participate in repopulating damaged parenchyma (Shao et al., 2022; Han et al., 2019; Lin et al., 2022). Functionally, SOX9 acts downstream of YAP and promotes hepatocyte proliferation through upregulation of TGF-α, thereby facilitating liver regeneration (Liu et al., 2025; Ding and Sancho-Bru, 2022). Conversely, hepatocyte-specific *Sox9* deletion alleviates acute injury (Qin et al., 2023), while *Sox9* overexpression activates AMPK signaling, reducing lipid accumulation in metabolic steatohepatitis but also promoting fibrosis (Manco et al., 2019; Trogisch et al., 2024; Athwal et al., 2018). These findings suggest that SOX9^+^ hepatocytes possess dual and context-dependent roles—protective during regeneration yet potentially maladaptive under chronic injury (Shang et al., 2024).

Recent advances in single-cell RNA sequencing (scRNA-seq) have enabled the precise identification of distinct hepatocyte subpopulations and the dissection of their functional heterogeneity at single-cell resolution (Merrell et al., 2021; Brazovskaja et al., 2024). Leveraging this approach, we integrated multiple scRNA-seq datasets from regenerative, acute, and chronic liver injury models to systematically characterize the distribution, transcriptional features, and functional pathways of SOX9^+^ hepatocytes. Our comprehensive analysis provides new insight into hepatocyte plasticity and elucidates the dynamic contributions of SOX9^+^ hepatocytes to both liver regeneration and pathological remodeling.

## 2 Materials and methods

### 2.1 Data sources

Publicly available single-cell RNA sequencing (scRNA-seq) datasets were obtained from the GEO and CNGB databases, including the CNGB Nucleotide Sequence Archive (CNP0002310) (Xu et al., 2024), Zenodo repository (Zenodo.6035873) (Ben-Moshe et al., 2022), and GEO accessions (GSE202379) (Gribben et al., 2024). All datasets were generated using the 10x Genomics Chromium platform.

### 2.2 Preprocessing and quality control

Data analysis was performed using Seurat v5 in R. Highly variable genes (top 2000) were identified using the FindVariableFeatures function, followed by principal component analysis (PCA). Batch effects were corrected using the Harmony package prior to clustering. Cells were clustered with FindNeighbors and FindClusters. Filtering thresholds were set at nFeature_RNA >500 and percent. mt <10% to exclude low-quality cells and potential doublets.

### 2.3 Identification and annotation of SOX9^+^ hepatocytes

Cell-type annotation was performed using established hepatocyte and biliary marker genes to identify hepatocyte clusters. Within annotated hepatocyte clusters, cells with detectable Sox9 expression (expression value >0) were defined as SOX9^+^ hepatocytes, whereas cells lacking detectable Sox9 expression were classified as SOX9^-^ hepatocytes. The proportion of SOX9^+^ hepatocytes was calculated for each sample and time point.

### 2.4 Analysis of SOX9^+^ hepatocytes

Differentially expressed genes (DEGs) between SOX9^+^ and SOX9^-^ hepatocytes were identified using *FindMarkers* with the criteria |log_2_FC| >0.25 and FDR <0.05. Marker genes of SOX9^+^ hepatocytes were determined based on DEG analysis. Gene Set Enrichment Analysis (GSEA) for KEGG pathways was performed on all SOX9^+^ hepatocytes in each experimental model.

### 2.5 Temporal dynamics

All SOX9^+^ hepatocytes were extracted and re-clustered within each model to identify distinct subpopulations and to characterize their transcriptional changes over time and across disease conditions. KEGG enrichment analysis was performed for each SOX9^+^ subcluster.

Temporal changes in the proportions of SOX9^+^ hepatocytes were quantified and visualized to delineate their activation kinetics during liver regeneration.

## 3 Results

### 3.1 Transcriptional heterogeneity and dynamic remodeling of SOX9^+^ hepatocytes during liver regeneration after partial hepatectomy

**FIGURE 1:**
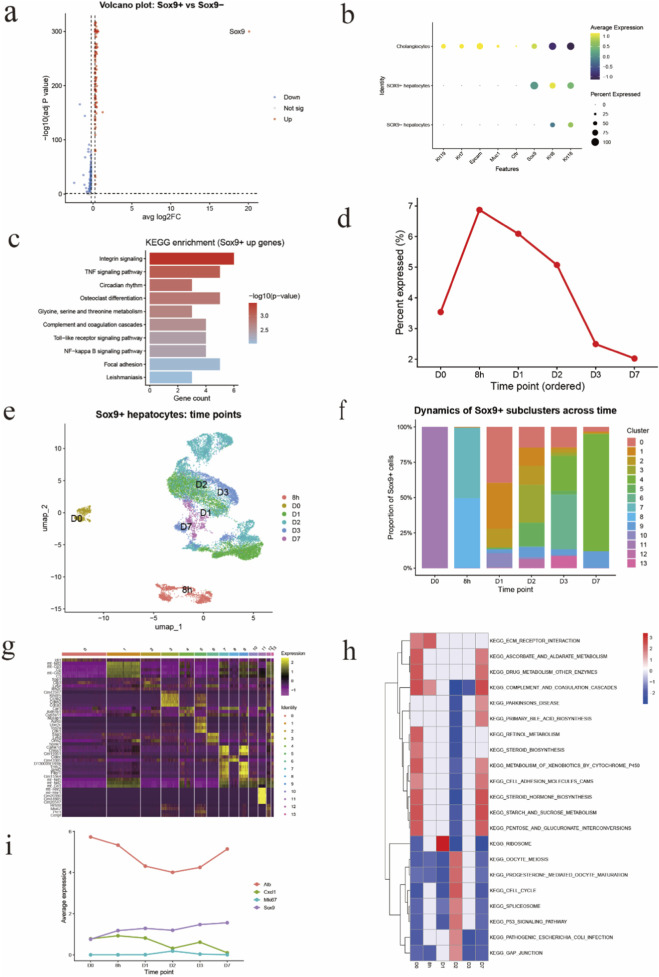
Transcriptional heterogeneity and dynamic remodeling of SOX9^+^ hepatocytes during liver regeneration after partial hepatectomy **(a)** Volcano plot showing differentially expressed genes between SOX9^+^ and SOX9^-^ hepatocytes following partial hepatectomy. Each dot represents one gene. Genes significantly upregulated in SOX9^+^ hepatocytes are shown in red, downregulated genes in blue, and non-significant genes in gray. The dashed line indicates the statistical significance threshold. **(b)** Dot plot showing the expression of representative marker genes across hepatocyte and cholangiocyte populations. Dot size indicates the percentage of cells expressing each gene, and color intensity represents the average expression level. SOX9^+^ hepatocytes retain expression of hepatocyte markers while lacking classical cholangiocyte markers, supporting their hepatocyte lineage identity. **(c)** Kyoto Encyclopedia of Genes and Genomes (KEGG) pathway enrichment analysis of genes upregulated in SOX9^+^ hepatocytes. Bar length represents the number of genes enriched in each pathway, and color intensity indicates statistical significance (–log10 *P* value). Enriched pathways are primarily associated with inflammatory signaling, immune responses, and cell–matrix interactions. **(d)** Temporal dynamics of SOX9^+^ hepatocytes following partial hepatectomy. The proportion of SOX9^+^ hepatocytes among total hepatocytes is shown across post-operative time points, demonstrating a transient expansion during early regeneration followed by a decline at later stages. **(e)** Uniform Manifold Approximation and Projection (UMAP) visualization of SOX9^+^ hepatocytes colored by post-hepatectomy time points. Cells from different time points occupy distinct regions in low-dimensional space, indicating pronounced temporal transcriptional remodeling of SOX9^+^ hepatocytes during regeneration. **(f)** Stacked bar plot showing the relative composition of SOX9^+^ hepatocyte subclusters across post-hepatectomy time points. The proportion of individual subclusters changes dynamically over time, revealing progressive reorganization of SOX9^+^ hepatocyte states during regeneration. **(g)** Heatmap of scaled expression levels for representative marker genes across SOX9^+^ hepatocyte subclusters. Rows represent genes and columns represent subclusters. Distinct gene expression patterns define transcriptionally heterogeneous SOX9^+^ hepatocyte states. **(h)** KEGG pathway enrichment heatmap for SOX9^+^ hepatocyte subclusters across time points. Colors indicate normalized enrichment scores (NES), with red representing positive enrichment and blue representing negative enrichment. Early time points are enriched for metabolic pathways, whereas later stages show enrichment of pathways related to cell cycle regulation, cell adhesion, and tissue remodeling. **(i)** Line plots showing average expression of representative genes (*Alb*, *Sox9*, *Cxcl1*, and *Mki67*) across post-hepatectomy time points. These genes illustrate stepwise functional reprogramming of SOX9^+^ hepatocytes, including reduced hepatocyte metabolic gene expression, sustained SOX9 expression, and transient induction of inflammatory and proliferative markers during regeneration.

To characterize SOX9^+^ hepatocytes during liver regeneration after partial hepatectomy, we first compared the transcriptomes of SOX9^+^ and SOX9^-^ hepatocytes. Differential gene expression analysis revealed widespread transcriptional differences between the two populations, as visualized by a volcano plot (Figure 1a). A substantial number of genes were significantly upregulated in SOX9^+^ hepatocytes, indicating that these cells adopt a transcriptional program distinct from SOX9^-^ hepatocytes rather than representing minor transcriptional variation within the hepatocyte compartment.

We next examined the expression of representative lineage and functional markers across hepatocyte populations to define the cellular identity of SOX9^+^ hepatocytes. Dot plot analysis demonstrated that SOX9^+^ hepatocytes continued to express canonical hepatocyte markers, confirming their hepatocyte lineage origin (Figure 1b). Importantly, these cells did not express classical cholangiocyte markers, excluding biliary contamination. In contrast to SOX9^-^ hepatocytes, SOX9^+^ hepatocytes displayed increased expression of genes associated with progenitor-like and stress-responsive states, suggesting that SOX9^+^ hepatocytes represent a distinct hepatocyte state induced during regeneration rather than a separate epithelial lineage.

To gain functional insight into the SOX9^+^ hepatocyte state, we performed KEGG pathway enrichment analysis on genes upregulated in SOX9^+^ hepatocytes. This analysis revealed significant enrichment of pathways related to inflammatory and immune signaling, including TNF, NF-κB, and Toll-like receptor signaling pathways, as well as pathways associated with integrin signaling and focal adhesion (Figure 1c). These enriched pathways suggest that SOX9^+^ hepatocytes actively engage in inflammatory signaling and cell–matrix interactions, processes known to be critical for effective tissue remodeling and regeneration following liver injury.

We also investigated the temporal dynamics of SOX9^+^ hepatocytes following partial hepatectomy. Quantification of SOX9^+^ hepatocytes across post-operative time points revealed a rapid increase in their proportion during early regenerative stages, followed by a gradual decline as regeneration progressed toward completion (Figure 1d). This transient expansion indicates that SOX9^+^ hepatocytes represent a regeneration-associated hepatocyte population that emerges in response to surgical injury and diminishes as liver homeostasis is restored.

To further resolve the temporal organization of SOX9^+^ hepatocytes during liver regeneration, we visualized SOX9^+^ hepatocytes across post-hepatectomy time points using UMAP embedding (Figure 1e). SOX9^+^ hepatocytes segregated into distinct regions corresponding to different time points, with minimal overlap between early (8 h, D0) and later regenerative stages (D1–D7). This temporal separation indicates pronounced transcriptional remodeling of SOX9^+^ hepatocytes as regeneration progresses, rather than persistence of a static SOX9^+^ cell population. Notably, SOX9^+^ hepatocytes from early time points occupied discrete UMAP regions, whereas cells from later stages showed increased heterogeneity, suggesting diversification of SOX9^+^ hepatocyte states during regenerative progression.

To quantify the temporal dynamics of SOX9^+^ hepatocyte heterogeneity, we examined the composition of SOX9^+^ subclusters across post-hepatectomy time points. The relative abundance of individual SOX9^+^ subclusters changed markedly over time (Figure 1f). Early after hepatectomy, SOX9^+^ hepatocytes were dominated by a limited number of subclusters, whereas later stages exhibited a broader and more complex subcluster composition. This dynamic redistribution indicates that SOX9^+^ hepatocytes undergo progressive state transitions during regeneration, with distinct subpopulations emerging and resolving at different regenerative stages.

To characterize the molecular features underlying SOX9^+^ hepatocyte heterogeneity, we identified marker genes for each SOX9^+^ subcluster. Heatmap visualization revealed clear transcriptional differences among subclusters, with distinct gene expression patterns defining individual states (Figure 1g). Several subclusters were characterized by enrichment of genes associated with stress responses, inflammatory signaling, and cell–matrix interaction, whereas others displayed higher expression of metabolic or housekeeping genes. These findings demonstrate that SOX9^+^ hepatocytes comprise multiple transcriptionally distinct states rather than a homogeneous population.

To link transcriptional heterogeneity to functional differences, we performed KEGG pathway enrichment analysis for SOX9^+^ hepatocyte subclusters across time points (Figure 1h). Early regenerative stages were enriched for metabolic pathways, including bile acid metabolism and xenobiotic metabolism, consistent with retention of hepatocyte functional programs. In contrast, later stages exhibited enrichment of pathways related to cell cycle regulation, p53 signaling, ECM–receptor interaction, and cell adhesion, indicating activation of proliferative and tissue remodeling programs. These pathway shifts suggest a temporal transition of SOX9^+^ hepatocytes from metabolically active states toward proliferative and remodeling-associated states during regeneration.

To further illustrate temporal functional changes, we examined the expression dynamics of representative genes across post-hepatectomy time points. Expression of the hepatocyte metabolic marker *Alb* decreased during early regenerative stages and partially recovered at later time points (Figure 1i). In contrast, expression of *Sox9* increased progressively, consistent with sustained progenitor-associated identity during regeneration. Inflammatory and proliferative markers, including *Cxcl1* and *Mki67*, exhibited transient induction at early to intermediate stages, coinciding with peak regenerative activity. These gene expression dynamics reinforce the concept that SOX9^+^ hepatocytes undergo stepwise functional reprogramming during liver regeneration.

### 3.2 Temporal trajectories of SOX9^+^ hepatocytes after acetaminophen (APAP) injury

**FIGURE 2:**
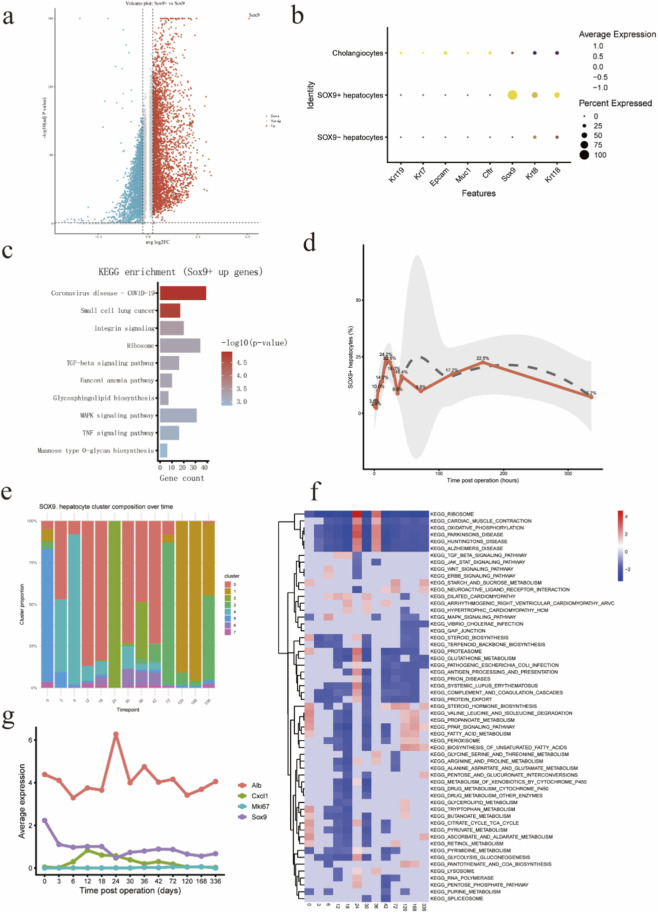
Transcriptional reprogramming and dynamic heterogeneity of SOX9^+^ hepatocytes following APAP-induced liver injury **(a)** Volcano plot showing differentially expressed genes between SOX9^+^ and SOX9^-^ hepatocytes following acetaminophen (APAP)–induced liver injury. Each dot represents one gene. Genes significantly upregulated in SOX9^+^ hepatocytes are shown in red, downregulated genes in blue, and non-significant genes in gray. The dashed lines indicate thresholds for statistical significance and log_2_ fold change. SOX9 is highlighted as a representative marker. **(b)** Dot plot showing the expression of representative marker genes across cholangiocytes, SOX9^+^ hepatocytes, and SOX9^-^ hepatocytes. Dot size indicates the percentage of cells expressing each gene, and color intensity represents the average expression level. SOX9^+^ hepatocytes retain expression of hepatocyte-associated markers while lacking classical cholangiocyte markers (e.g., *Krt19*), indicating that SOX9^+^ hepatocytes represent a distinct hepatocyte state rather than biliary cells. **(c)** KEGG pathway enrichment analysis of genes upregulated in SOX9^+^ hepatocytes following APAP injury. Bar length represents the number of genes enriched in each pathway, and color intensity indicates statistical significance (–log_10_ *P* value). Enriched pathways include inflammatory signaling, integrin signaling, and stress-associated pathways, consistent with an injury-responsive hepatocyte state. **(d)** Temporal dynamics of SOX9^+^ hepatocytes following APAP-induced liver injury. The proportion of SOX9^+^ hepatocytes among total hepatocytes is shown across time points post injury. SOX9^+^ hepatocytes exhibit a rapid increase during the acute injury phase, followed by a gradual decline during the recovery phase, indicating a transient, injury-associated expansion. **(e)** Stacked bar plot showing the relative composition of SOX9^+^ hepatocyte subclusters across time points following APAP injury. The proportion of individual subclusters changes dynamically over time, revealing substantial remodeling of SOX9^+^ hepatocyte states during injury and recovery. **(f)** Heatmap showing KEGG pathway enrichment dynamics of SOX9^+^ hepatocytes across time points following APAP-induced liver injury. Colors represent normalized enrichment scores (NES), with red indicating positive enrichment and blue indicating negative enrichment. Early time points are characterized by enrichment of stress and inflammatory pathways, whereas later stages show reactivation of metabolic pathways, reflecting resolution of injury and functional recovery. **(g)** Line plots showing average expression of representative genes across time points following APAP injury. Expression of the hepatocyte metabolic marker *Alb* decreases during the acute injury phase and partially recovers during later stages. In contrast, *Sox9* expression is maintained or increased, while inflammatory and proliferative markers (*Cxcl1* and *Mki67*) show transient induction, highlighting stepwise functional reprogramming of SOX9^+^ hepatocytes during injury and repair.

To characterize SOX9^+^ hepatocytes in the context of APAP, we compared the transcriptional profiles of SOX9^+^ and SOX9^-^ hepatocytes. Differential expression analysis revealed extensive transcriptional differences between the two populations, as visualized by a volcano plot (Figure 2a). A large number of genes were significantly upregulated in SOX9^+^ hepatocytes, indicating that these cells adopt a distinct transcriptional program in response to APAP-induced injury.

To determine the cellular identity of SOX9^+^ hepatocytes after APAP injury, we examined the expression of representative lineage markers across epithelial cell populations. Dot plot analysis demonstrated that SOX9^+^ hepatocytes continued to express canonical hepatocyte-associated genes, while lacking expression of classical cholangiocyte markers such as *Krt19* (Figure 2b). These results exclude biliary contamination and confirm that SOX9^+^ hepatocytes represent a hepatocyte-derived population that acquires a distinct transcriptional state following injury.

To gain insight into the functional programs associated with the SOX9^+^ hepatocyte state, we performed KEGG pathway enrichment analysis on genes upregulated in SOX9^+^ hepatocytes. This analysis revealed significant enrichment of pathways related to inflammatory and stress responses, including integrin signaling, TNF signaling, MAPK signaling, and other injury-associated pathways (Figure 2c). These findings indicate that SOX9^+^ hepatocytes engage inflammatory and stress-responsive signaling programs during APAP-induced liver injury.

We next examined the temporal dynamics of SOX9^+^ hepatocytes following APAP administration. Quantification of SOX9^+^ hepatocytes across time points revealed a rapid increase in their proportion during the acute injury phase, followed by a gradual decline during the recovery phase (Figure 2d). This transient expansion suggests that SOX9^+^ hepatocytes represent an injury-induced hepatocyte state that emerges rapidly in response to toxic injury and resolves as liver homeostasis is restored.

To further resolve heterogeneity within the SOX9^+^ hepatocyte population, we analyzed the composition of SOX9^+^ hepatocyte subclusters across time points following APAP injury. The relative abundance of individual subclusters changed markedly over time, with specific subclusters predominating during early injury stages and others emerging during later recovery phases (Figure 2e). These results demonstrate dynamic remodeling of SOX9^+^ hepatocyte states throughout the injury–recovery continuum.

To link transcriptional heterogeneity to functional differences, we performed KEGG pathway enrichment analysis across SOX9^+^ hepatocyte subclusters over time. Early time points following APAP injury were characterized by enrichment of stress and inflammatory pathways, whereas later stages showed gradual reactivation of metabolic pathways, consistent with injury resolution and functional recovery (Figure 2f). These results indicate a temporal shift in functional programs of SOX9^+^ hepatocytes during the course of injury and repair.

Finally, we examined the temporal expression patterns of representative genes to illustrate functional changes in SOX9^+^ hepatocytes following APAP injury. Expression of the hepatocyte metabolic marker *Alb* decreased during the acute injury phase and partially recovered at later time points. In contrast, *Sox9* expression was maintained or modestly increased, while inflammatory and proliferative markers such as *Cxcl1* and *Mki67* exhibited transient induction during early to intermediate stages (Figure 2g). These expression dynamics highlight stepwise functional reprogramming of SOX9^+^ hepatocytes during acute injury and subsequent repair.

### 3.3 Progressive accumulation and transcriptional reprogramming of SOX9^+^ hepatocytes during metabolic liver disease

**FIGURE 3:**
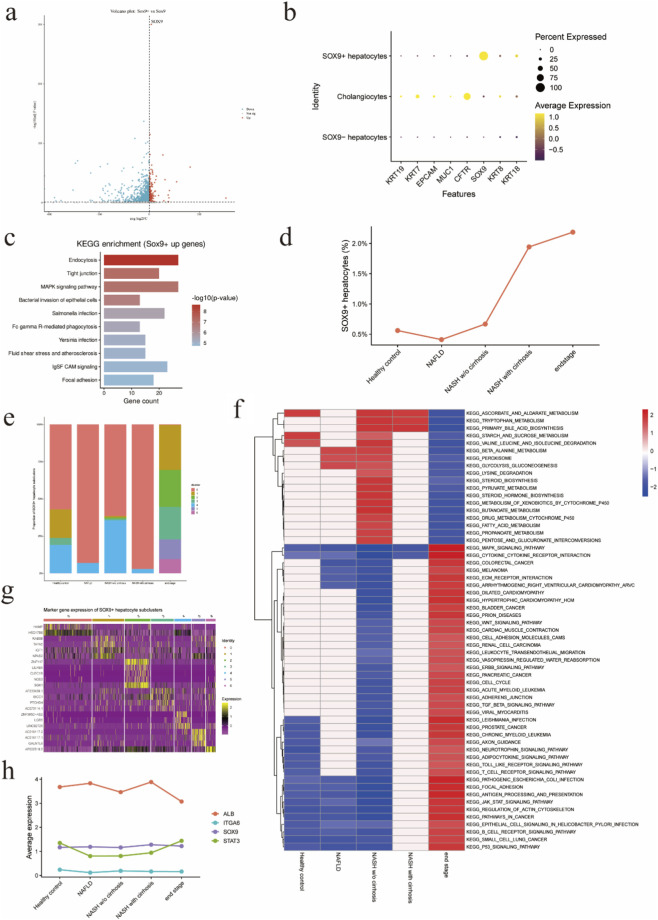
Transcriptional reprogramming and dynamic heterogeneity of SOX9^+^ hepatocytes during chronic liver disease progression. **(a)** Volcano plot showing differentially expressed genes between SOX9^+^ and SOX9^−^ hepatocytes. Each dot represents one gene. Genes significantly upregulated in SOX9^+^ hepatocytes are shown in red, downregulated genes in blue, and non-significant genes in gray. Dashed lines indicate thresholds for log_2_ fold change and statistical significance. *SOX9* is highlighted as a representative marker gene. **(b)** Dot plot showing the expression of representative marker genes across cholangiocytes, SOX9^+^ hepatocytes, and SOX9^−^ hepatocytes. Dot size indicates the percentage of cells expressing each gene, and color intensity represents average expression levels. SOX9^+^ hepatocytes express hepatocyte-associated markers while lacking classical cholangiocyte markers such as *KRT19*, confirming their hepatocyte lineage identity and excluding biliary contamination. **(c)** KEGG pathway enrichment analysis of genes upregulated in SOX9^+^ hepatocytes. Bar length represents the number of genes associated with each pathway, and color intensity indicates statistical significance ( -log_10_ P value). Enriched pathways include inflammatory signaling, stress-response pathways, and cell -matrix interaction -related processes. **(d)** Proportion of SOX9^+^ hepatocytes across disease stages. The percentage of SOX9^+^ hepatocytes among total hepatocytes is shown for healthy controls, NAFLD, NASH without cirrhosis, NASH with cirrhosis, and end-stage disease. SOX9^+^ hepatocytes exhibit a stage-dependent increase, with a pronounced expansion in advanced disease stages. **(e)** Stacked bar plot showing the relative composition of SOX9^+^ hepatocyte subclusters across disease stages. Each bar represents one disease stage, and colors indicate distinct SOX9^+^ subclusters. The distribution of subclusters changes progressively during disease progression, revealing dynamic remodeling of SOX9^+^ hepatocyte states. **(f)** Heatmap showing KEGG pathway enrichment dynamics across disease stages. Colors represent normalized enrichment scores (NES), with red indicating positive enrichment and blue indicating negative enrichment. Early disease stages are enriched for metabolic pathways, whereas advanced stages show enrichment of inflammatory, proliferative, and cell -matrix interaction -related pathways, indicating a functional transition of SOX9^+^ hepatocytes during disease progression. **(g)** Heatmap showing scaled expression of representative marker genes across SOX9^+^ hepatocyte subclusters. Rows represent genes and columns represent subclusters. Distinct gene expression patterns define transcriptionally heterogeneous SOX9^+^ hepatocyte subpopulations. **(h)** Line plots showing average expression of representative genes across disease stages. Expression of the hepatocyte metabolic marker *ALB* decreases with disease progression, whereas *SOX9* expression is maintained. Inflammatory and signaling-associated genes (*STAT3*) and ECM/adhesion-related genes (*ITGA6*) increase in advanced disease stages, illustrating stepwise functional reprogramming of SOX9^+^ hepatocytes.

We compared the transcriptomic profiles of SOX9^+^ and SOX9^-^ hepatocytes to characterize SOX9^+^ hepatocytes in metabolic liver disease. Distinct gene expression patterns between the two groups are shown in the volcano plot (Figure 3a). To clarify the cellular identity of SOX9^+^ hepatocytes, we examined the expression of representative hepatocyte and cholangiocyte marker genes. Dot plot analysis showed that SOX9^+^ hepatocytes expressed hepatocyte-associated markers while lacking expression of classical cholangiocyte markers such as *KRT19* (Figure 3b). In contrast, cholangiocytes displayed robust expression of biliary markers.

To gain insight into the functional programs associated with SOX9^+^ hepatocytes, we performed KEGG pathway enrichment analysis on genes upregulated in SOX9^+^ hepatocytes. This analysis revealed significant enrichment of pathways related to inflammatory signaling and stress responses, including MAPK signaling, TNF signaling, bacterial invasion–related pathways, and focal adhesion (Figure 3c). These findings suggest that SOX9^+^ hepatocytes engage stress-responsive and cell–matrix interaction programs, consistent with a non-homeostatic hepatocyte state.

We next quantified the proportion of SOX9^+^ hepatocytes across disease stages. SOX9^+^ hepatocytes were rare in healthy controls and NAFLD, accounting for less than 1% of hepatocytes. Their proportion increased modestly in NASH without cirrhosis and exhibited a pronounced expansion in NASH with cirrhosis and end-stage disease, reaching approximately 2% of the hepatocyte population (Figure 3d). These results indicate a stage-dependent accumulation of SOX9^+^ hepatocytes during liver disease progression, with a sharp increase coinciding with advanced disease stages.

To further resolve heterogeneity within the SOX9^+^ hepatocyte population, we examined the composition of SOX9^+^ hepatocyte subclusters across disease stages. Stacked bar plots revealed substantial shifts in subcluster composition during disease progression (Figure 3e). Early disease stages were dominated by a limited number of subclusters, whereas advanced stages showed increased representation of additional subclusters, indicating progressive remodeling of SOX9^+^ hepatocyte states during disease progression.

We next characterized the transcriptional features underlying SOX9^+^ hepatocyte heterogeneity by identifying marker genes for each subcluster. Heatmap analysis demonstrated clear differences in gene expression patterns among SOX9^+^ hepatocyte subclusters (Figure 3g). Distinct subsets of genes defined individual subclusters, indicating that SOX9^+^ hepatocytes comprise multiple transcriptionally distinct states rather than a homogeneous population.

To investigate functional changes associated with disease progression, we analyzed KEGG pathway enrichment dynamics across disease stages. Early stages were characterized by enrichment of metabolic pathways, including amino acid metabolism, fatty acid metabolism, and xenobiotic metabolism (Figure 3f). In contrast, advanced disease stages showed strong enrichment of pathways related to inflammatory signaling, cytokine–cytokine receptor interaction, cell cycle regulation, ECM–receptor interaction, and cancer-associated pathways, indicating a functional transition toward inflammatory, proliferative, and tissue-remodeling programs.

Finally, we examined the expression dynamics of representative genes across disease stages to illustrate functional reprogramming of SOX9^+^ hepatocytes. Expression of the hepatocyte metabolic marker *ALB* progressively decreased with disease severity, whereas *SOX9* expression was maintained. In parallel, inflammatory and signaling-associated genes such as *STAT3* and ECM-related genes such as *ITGA6* were upregulated in advanced disease stages (Figure 3h). These expression patterns highlight a progressive shift of SOX9^+^ hepatocytes from a metabolically active hepatocyte state toward an inflammatory and tissue-interactive phenotype during disease progression.

## 4 Discussion

This study provides a comprehensive characterization of SOX9^+^ hepatocyte dynamics under both acute regenerative and chronic pathological conditions. Through integrated single-cell transcriptomic analyses across multiple liver injury models, we demonstrate that SOX9^+^ hepatocytes serve as a plastic interface between hepatocyte regeneration and ductular transformation, adapting their transcriptional programs to the nature and duration of liver injury. Across three representative models—partial hepatectomy (PHx), acetaminophen (APAP)-induced hepatotoxicity, and non-alcoholic fatty liver disease (NAFLD) progression—SOX9^+^ hepatocytes exhibited a unifying pattern of context-dependent activation and functional reprogramming. Across the different injury models, the proportion of SOX9^+^ hepatocytes exhibited distinct dynamic patterns. In the APAP-induced injury model, SOX9^+^ hepatocytes showed the largest overall expansion, with their proportion reaching up to approximately 20%, indicating a robust response to acute toxic injury. In contrast, following partial hepatectomy (PHx), the increase in SOX9^+^ hepatocytes occurred more rapidly, with an approximately threefold elevation detectable as early as 8 h after surgery, consistent with the immediate regenerative demand. During NAFLD progression, the proportion of SOX9^+^ hepatocytes increased in a disease severity–dependent manner, suggesting a gradual and sustained induction associated with chronic metabolic injury. Despite distinct etiologies and temporal kinetics, these cells consistently shared transcriptional signatures enriched in stress response, metabolic adaptation, and cellular plasticity, indicating a conserved adaptive mechanism that bridges regeneration and pathology (Cao et al., 2017).

SOX9 expression has been widely reported in multiple hepatic cell populations, including cholangiocytes, hepatic progenitor cells, and immature or hybrid hepatocytes, particularly under conditions of liver injury and regeneration (Shao et al., 2022; Lin et al., 2022; Shang et al., 2024). These SOX9^+^ hepatocytes exhibited distinct transcriptional programs compared with other SOX9^-^ hepatocytes, as supported by differential gene expression and pathway enrichment analyses. Notably, pathway analyses revealed enrichment of signaling pathways related to cellular activation, adhesion, and stress responses, suggesting that SOX9^+^ hepatocytes represent a functionally specialized hepatocyte subpopulation rather than a generic progenitor state. While SOX9^+^ hepatocytes constituted a minor fraction in healthy and early NAFLD livers, their proportion increased markedly during liver regeneration, cirrhosis, and end-stage liver disease, indicating stage-specific expansion or induction. This dynamic behavior suggests that SOX9^+^ hepatocytes may adopt distinct functional roles depending on pathological context, potentially reflecting adaptive responses to chronic injury, altered tissue architecture, or inflammatory microenvironments.

In the PHx model, SOX9^+^ hepatocytes underwent a rapid but transient activation phase, characterized by short-term enrichment of MAPK and PI3K–Akt signaling pathways, followed by restoration of metabolic equilibrium. These results are consistent with cellular reprogramming that balances the metabolic and proliferative demands of the regenerating liver. (Chembazhi et al., 2021).This dynamic suggests that SOX9^+^ hepatocytes represent a metabolically responsive subpopulation that transiently supports early regenerative metabolism. Consistent with our findings, previous studies have reported that *Sox9* expression rises markedly within 3 h after hepatectomy, underscoring its crucial role in acute regeneration (Shao et al., 2022; Han et al., 2019). Moreover, hepatocyte-specific *Sox9* knockout was shown to delay regeneration, confirming that SOX9^+^ hepatocytes are essential for initiating and sustaining early repair processes (Liu et al., 2025).

In the APAP-induced injury model, SOX9^+^ hepatocytes displayed biphasic transcriptional dynamics, aligning with their dual roles in the injury and recovery phases. Initially, these cells activated oxidative phosphorylation and cytokine-related pathways, facilitating early stress response and survival under hepatotoxic conditions. During recovery, they reactivated lipid metabolism and energy-related processes, reflecting a return to metabolic homeostasis. At the gene expression level, SOX9^+^ hepatocytes exhibit dynamic modulation of hepatocyte functional markers, including Alb, alongside transient changes in proliferation- and stress-associated genes. Notably, while Alb expression fluctuates over time, it is not lost, indicating preservation of hepatocyte identity during the regenerative process. This pattern suggests that SOX9 expression marks a plastic and inducible hepatocyte state that enables reversible functional adaptation to injury rather than a terminally altered phenotype. Importantly, although our data define the transcriptional characteristics and temporal dynamics of SOX9^+^ hepatocytes, the precise cellular origin and lineage relationships of this population cannot be determined from the current analyses. Whether SOX9^+^ hepatocytes arise from pre-existing hepatocytes, progenitor-like cells, or alternative cellular sources will require further investigation using lineage tracing and functional approaches.

By contrast, during NAFLD progression, SOX9^+^ hepatocytes exhibited progressive expansion accompanied by the acquisition of inflammatory, angiogenic, and wound-healing transcriptional programs (Ben-Moshe et al., 2022). Functional enrichment analysis revealed a stage-dependent shift from lipid metabolic processes in early NAFLD and NASH to immune and stress-response pathways in cirrhosis and end-stage disease. This gradual transition suggests that chronic metabolic stress drives the conversion of SOX9^+^ hepatocytes from a metabolic to an inflammatory phenotype, potentially fostering fibrogenesis and the development of tumor-promoting microenvironments (Wang et al., 2020). These findings align with previous reports that *Sox9* acts as a key fibrogenic factor and marker of the ductular reaction (Li et al., 2025). Furthermore, sustained SOX9 expression has been linked to hepatocyte dedifferentiation during chronic liver injury, supporting the view that prolonged SOX9 activation contributes to pathological remodeling rather than regeneration (Lin et al., 2022).

Collectively, our results suggest that SOX9^+^ hepatocytes form a continuum of adaptive states—acting as metabolic buffers during regeneration, stress responders during acute injury, and inflammatory mediators during chronic disease. This continuum highlights their dualistic nature: protective and regenerative in the short term, yet maladaptive when persistently activated. Such plasticity underscores their pivotal role in linking regenerative repair to disease progression, bridging the molecular landscapes of recovery and fibrosis.

In conclusion, this study establishes SOX9^+^ hepatocytes as a central hub of liver plasticity. Transient activation of SOX9 promotes hepatocyte proliferation and tissue restoration following acute injury, whereas sustained activation in chronic conditions drives ductular metaplasia, inflammation, and fibrosis. The balance between these two states determines whether hepatocyte reprogramming results in regeneration or pathological remodeling. These findings not only deepen our understanding of hepatocyte plasticity but also provide a conceptual framework for developing therapeutic strategies aimed at modulating SOX9 signaling to enhance liver repair while preventing fibrogenesis.

## Data Availability

The original contributions presented in the study are included in the article/supplementary material, further inquiries can be directed to the corresponding authors.
